# Tobacco outlet availability in Dutch rural and urban areas: Tessa van Deelen

**DOI:** 10.1093/eurpub/ckac130.219

**Published:** 2022-10-25

**Authors:** T van Deelen, S Belmonte, B van denPutte, AE Kunst, MAG Kuipers

**Affiliations:** 1 Public and Occupational Health, Amsterdam UMC Location University of Amsterdam, Amsterdam, Netherlands; 2 Amsterdam School of Communication Research, University of Amsterdam, Amsterdam, Netherlands

## Abstract

**Background:**

The Netherlands aims to reduce the availability of tobacco outlets by implementing a sales ban for vending machines (2022) and supermarkets (2024). The government intends to further phase out tobacco sales by petrol stations and small outlets after 2030. This study aims to understand its impact on tobacco outlet availability in the Netherlands, with particular attention to rural areas.

**Methods:**

An audit of tobacco retailers was held between Sept 2019-June 2020 in four cities (Amsterdam, Eindhoven, Haarlem, and Zwolle) and between March-Apr 2022 in seven rural municipalities (Aa en Hunze, Ermelo, Dinkelland, Montferland, Simpelveld, Veere, and Waadhoeke). Each identified retailer was visited and mapped using Global Positioning System (GPS). Tobacco outlet availability was calculated in terms of density per population and km2, and residents’ proximity to the nearest outlet.

**Results:**

In the rural areas, we found a total of 98 tobacco outlets, of which supermarkets (n = 57), petrol stations (26), small outlets (13) and tobacco specialist shops (2). In the four cities, we found a total of 870 outlets. Tobacco outlet density was much lower in rural areas than the four cities: 0.09 vs. 2.2 per km2 and 5.05 vs. 6.2 per 10,000 capita. The average shortest distance from an address to a tobacco outlet was much higher in rural areas (1.23km) compared to cities (0.31km). After implementation of all sales bans, tobacco outlet availability will reduce to 2 outlets in rural areas and 61 in urban areas which represents 0.1 and 0.4 per 10,000 capita, respectively. The distance will increase in cities (to 1.42km), but particularly in rural areas (to 5.28km) where 5 of the 7 municipalities did no longer include a tobacco outlet.

**Conclusions:**

The proposed restrictions on tobacco sales will strongly decrease tobacco outlet availability, and might even disappear in some rural areas. These results call into question how the tobacco industry would respond to the proposed restriction.

**Key messages:**

- This study is the first ex-ante policy evaluation to assess the impact of current and future tobacco control policies at the POS on tobacco outlet availability in the Netherlands.
- The proposed restrictions on tobacco sales will strongly decrease tobacco outlet availability, and might even disappear in some rural areas.

